# Correction: A novel mechanism of regulation of the oncogenic transcription factor GLI3 by toll-like receptor signaling

**DOI:** 10.18632/oncotarget.28430

**Published:** 2023-05-12

**Authors:** Stephan J. Matissek, Mona Karbalivand, Weiguo Han, Ava Boutilier, Estefania Yzar-Garcia, Laura L. Kehoe, Devin Storm Gardner, Adam Hage, Krista Fleck, Vicki Jeffers, Ricardo Rajsbaum, Sherine F. Elsawa

**Affiliations:** ^1^Department of Molecular, Cellular and Biomedical Sciences, University of New Hampshire, Durham, NH, USA; ^2^Department of Microbiology and Immunology, University of Texas Medical Branch, Galveston, TX, USA; ^3^Institute for Human Infections and Immunity, University of Texas Medical Branch, Galveston, TX, USA

**This article has been corrected:** In Figure 3B, the beta actin western blot of U937 cells is an accidental duplicate of the western blot of MM6 cells. The correct Figure 3B, obtained using the original data, is shown below. The authors declare that these corrections do not change the results or conclusions of this paper.

Original article: Oncotarget. 2022; 13:944–959. 944-959. https://doi.org/10.18632/oncotarget.28261

**Figure 3:**
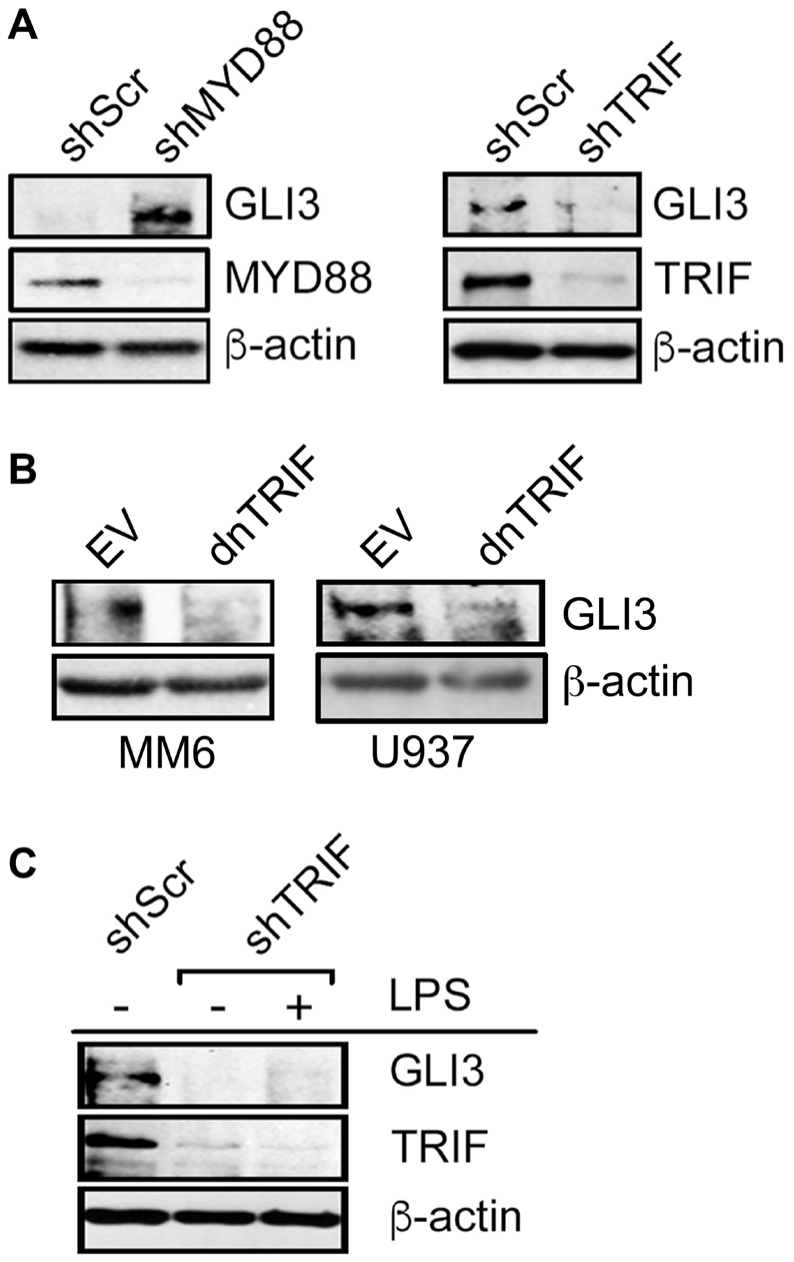
LPS-induced GLI3 is regulated by TRIF downstream of TLR4. (**A**) U937 cells (10 × 10^6^ cells) were transfected with 10 μg of either shMYD88, shTRIF or scrambled controls (shScr). After 2 days, cells were lysed and lysates were used to determine protein expression by western blot. (**B**) Monocytes (10 × 10^6^ cells) were transfected with a dominant negative form of TRIF (dnTRIF) or empty vector (Ctrl) for 2 days followed by determination of protein expression by western blot. (**C**) MM6 cells (10 × 10^6^ cells) were transfected with shTRIF or shScr and cultured for 30 h, followed by treatment with 100 ng/ml LPS. After an additional 12 h, cells were lysed and lysates were used for western blot to determine protein expression. These experiments were repeated at least 3 times with similar results.

