# Supplementary material for: A novel mechanism of regulation of the oncogenic transcription factor GLI3 by toll-like receptor signaling
Source: Oncotarget. 2022 Aug 3;13:944–59. doi: 10.18632/oncotarget.28261 (PMC9348707; doi:10.18632/oncotarget.28261)
Supplement: Supplementary file 1 [file oncotarget-13-28261-s001.pdf]

A novel mechanism of regulation of the oncogenic transcription factor GLI3 by toll-like receptor signaling

SUPPLEMENTARY MATERIALS

Differentiation of monocyte cell lines into macrophages

Human monocyte cell lines (MM6, THP-1 and U937) (2×10^6 cells/ml) were differentiated into macrophages by stimulation with PMA (5 ng/ml) for 24 hr, followed by washing and allowing cells to rest for an additional 24 hr. Cells were then stimulated with 100 ng/ml LPS for 1 hr and GLI3 expression was determined by qPCR.

Nuclear fractionation

THP-1 cells (10×10^6 cells) were treated with 1 mg/ml LPS for 24 hr. Cells were fractionated with NE-PER™ Nuclear and Cytoplasmic Extraction Reagents (Thermo Scientific) supplemented with 3% of 10× Pierce™ protease inhibitor (Thermo Scientific). Nuclear and cytoplasmic fractions were analyzed by western blot using the following antibodies: Lamin (nuclear; Cell Signaling Technology, #2032s), Tubulin (cytoplasmic; Cell signaling Technology, #3873s) and GLI3 (Novus AF3690) antibodies.

Generation and characterization of *M-Gli3*$^{-/-}$ mice

Mice lacking *Gli3* expression in myeloid cells were generated by crossing *Gli3*$^{fl/fl}$ mice (Jax stock # 008873) which harbor *loxP* sites flanking exon 8 of mouse *Gli3* with *LysM-cre*$^{+/+}$ mice (Jax stock # 004781). Mice genotyping is performed by PCR following recommendation on the Jackson Laboratory website.

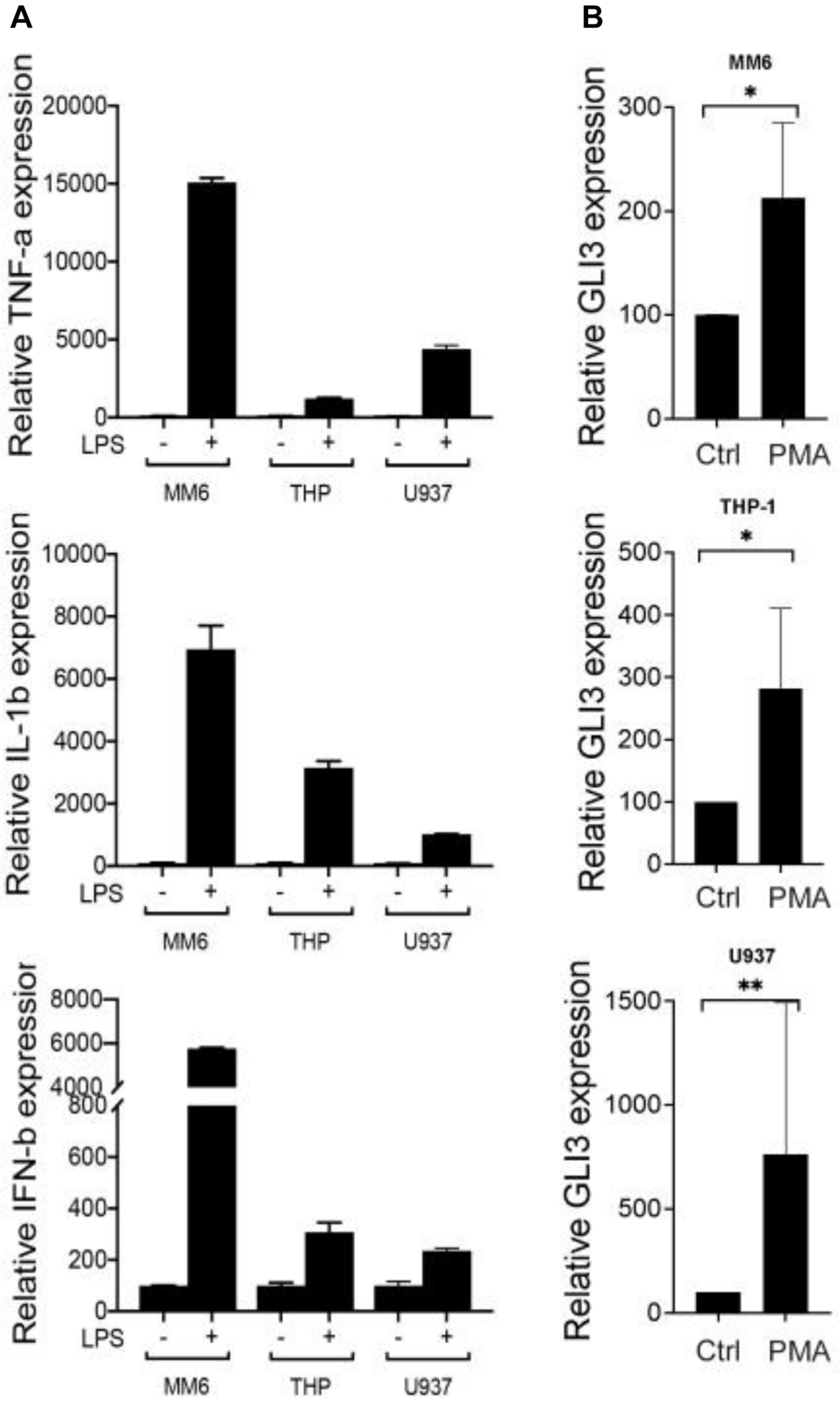

Supplementary Figure 1: Human monocyte cell lines are responsive to TLR4 stimulation. (**A**) Human monocyte cell lines (MM6, THP-1 and U937) (2×10^6 cells/ml) were treated with 100 ng/ml LPS for 1 hr. RNA was isolated and used to determine TNF-a, IL-1b and IFN-b expression by qPCR. (**B**) Monocyte cell lines (2×10^6 cells/ml) were differentiated into macrophages by stimulation with PMA (5 ng/ml) for 24 hr, followed by washing and allowing cells to rest for an additional 24 hr. Cells were then stimulated with 100 ng/ml LPS for 1 hr and GLI3 expression was determined by qPCR.

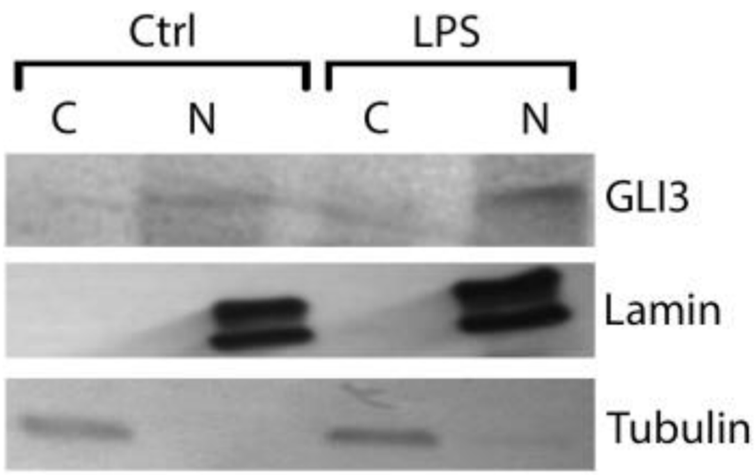

Supplementary Figure 2: Nuclear expression of GLI3. THP-1 cells (10×10^6 cells) were treated with 1 mg/ml LPS for 24 hr. Cells were fractioned with NE-PER™ Nuclear and Cytoplasmic Extraction Reagents (Thermo Scientific) supplemented with 3% of 10× Pierce TM protease inhibitor (Thermo Scientific). Nuclear and cytoplasmic fractions were analyzed by western blot.

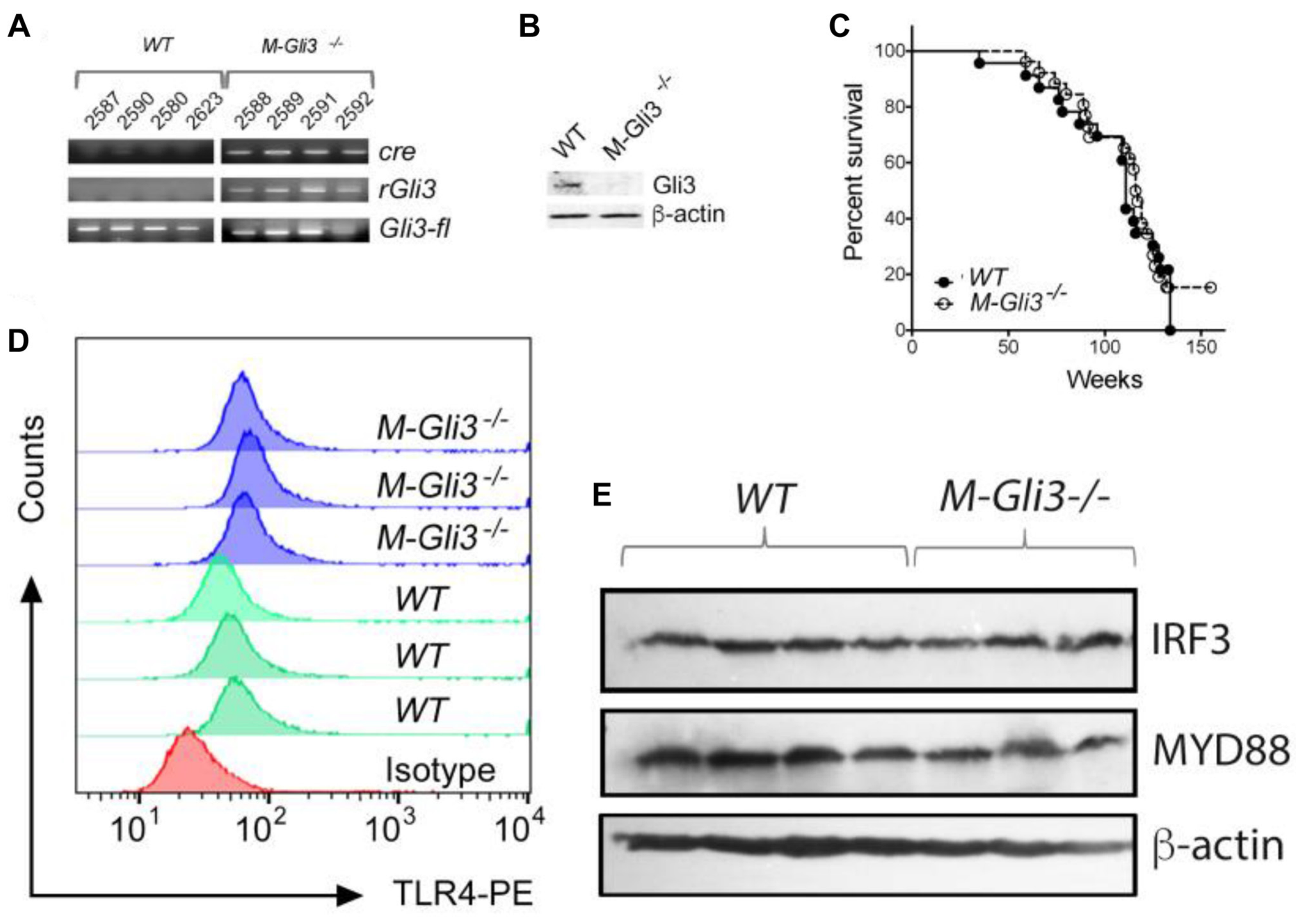

Supplementary Figure 3: Generation and characterization of *M-Gli3*$^{-/-}$ mice. (**A**) Mice are genotyped by PCR using primers specific for *Cre*, *Gli3fl/fl* (Gli3-fl) and the recombined *Gli3* (rGli3). Numbers represent individual mice identifiers in our colony. (**B**) Western blot analysis of GLI3 expression in IFA-elicited macrophages from *WT* and *M-Gli3*$^{-/-}$ mice. (**C**) Overall survival of *WT* and *M-Gli3*$^{-/-}$ mice. Mice survival was monitored for 150 weeks. (**D**) Macrophages from *WT* and *M-Gli3*$^{-/-}$ mice ($n = 3$ each) were stained with PE-conjugated TLR4 antibody followed by analysis on a FACS Calibur flow cytometer. (**E**) Macrophages from *WT* ($n = 4$) and *M-Gli3*$^{-/-}$ ($n = 3$) mice were lysed and used for western blot to determine IRF3 and MyD88 protein expression.

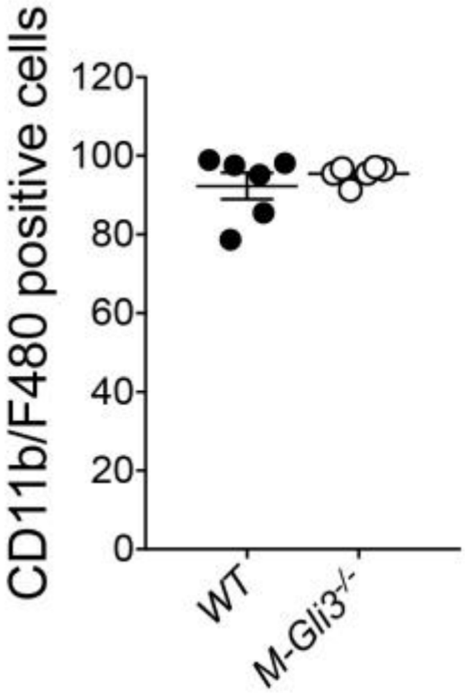

Supplementary Figure 4: Similar macrophages in *M-Gli3*$^{-/-}$ and WT mice. IFA-elicited macrophages from *WT* and *M-Gli3*$^{-/-}$ mice (n = 6 each) were isolated and allowed to adhere to cell culture plates for 1 hr followed by washing non-adherent cells. Cells were then harvested and stained with antibodies for the macrophage markers CD11b and F4/80 followed by analysis on a FACS Calibur flow cytometer.

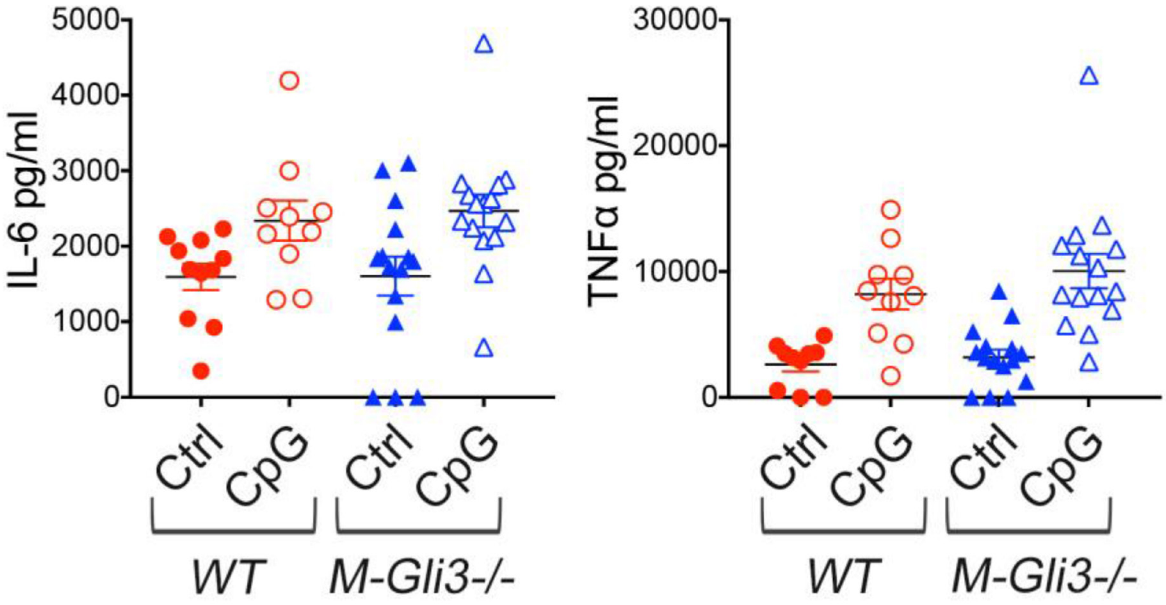

Supplementary Figure 5: MYD88 stimulation is not altered in *M-Gli3*$^{-/-}$ and WT mice. IFA-elicited macrophages from each mouse strain (n = 11 *WT*; n = 15 *M-Gli3*$^{-/-}$) were stimulated with 10 mg/ml CpG or left untreated for 24 hr. Supernatants were used to quantify cytokine levels by ELISA.

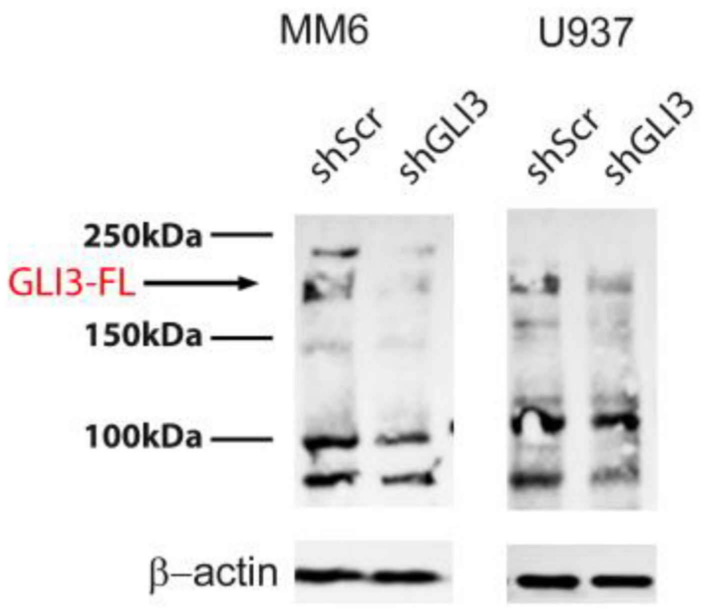

Supplementary Figure 6: Validation of GLI3 antibodies by western blot. Human monocyte cell lines (MM6 and U937) (4×10^6 cells/cuvette) were transfected by electroporation with shGLI3 or shScr. After 2 days, cells were lysed in western blot lysis buffer and analyzed by western blot to determine the expression of GLI3 (190 KDa).
